# Effects of information sources on public preventive behaviors in health emergencies: Evidence from a digital epidemiologic study during the COVID-19 pandemic

**DOI:** 10.3389/fpubh.2022.981649

**Published:** 2022-10-14

**Authors:** Benli Xue, Yibo Wu, Xiao Zheng, Yaqing Xue, Fang Dong, Shujuan Xiao, Mei Yin, Mingxu Wang, Yuxi Liu, Chichen Zhang

**Affiliations:** ^1^School of Health Management, Southern Medical University, Guangzhou, China; ^2^School of Public Health, Peking University, Beijing, China; ^3^Department of Health Management, Shunde Hospital, Southern Medical University (The First People's Hospital of Shunde, Foshan), Guangdong, China; ^4^School of Public Health, Southern Medical University, Guangzhou, China; ^5^School of Humanities and Social Sciences, Harbin Medical University, Harbin, China; ^6^School of Public Health, Xi'an Jiaotong University Health Science Center, Xi'an, China; ^7^Health Culture Research Center of Shaanxi, Xi'an, China; ^8^School of Humanities and Management, Guangdong Medical University, Dongguan, China; ^9^Department of Health Management, Nanfang Hospital, Southern Medical University, Guangzhou, China

**Keywords:** COVID-19, preventive behaviors, information sources, internet resources, public health

## Abstract

**Introduction:**

It has been approved that information sources would affect public behaviors. However, due to the outbreak of COVID-19, this influence was enhanced and showed a distinctive pattern among different populations, which has been less noticed before. We aimed to investigate the potential roles of different information sources in COVID-19 preventive behaviors of different publics.

**Methods:**

A cross-sectional online survey with 11,190 participants from 33 province-level regions in China was conducted during the COVID-19 pandemic. Sociodemographic characteristics, COVID-19 preventive behaviors, and information sources for COVID-19-related information were assessed. A mixed linear model was used to analyze risk factors of COVID-19 preventive behaviors. The effects of different information sources on COVID-19 prevention behaviors of different publics were analyzed.

**Results:**

Generally, the Chinese public had good COVID-19 preventive behaviors, and the top three COVID-19 preventive behaviors with the higher action rate were avoiding eat bushmeat (76.1%), a healthy diet (74.8%), and avoiding contact with people with symptoms of respiratory diseases (73.0%). About information sources, 12320 telephone (National Public Health Hotline) (−0.62, 95% CI: −0.94 to −0.31) and acquaintances consulting (−1.00, 95% CI: −1.31 to −0.69) were negatively associated with COVID-19 preventive behaviors, while internet resources, family doctors, hospitals, and community health centers were positively associated with COVID-19 preventive behaviors (1.00 vs. 0.47 vs. 0.46 vs. 0.33, *P* < 0.05). For older adults, accessing to COVID-19-related information through family doctors and community health centers were positively associated with COVID-19 preventive behaviors. For the non-educated, family doctors and community health centers had positive effects on their COVID-19 preventive behaviors. Family doctors and internet resources were positively associated with COVID-19 preventive behaviors among those earning 5,000 yuans and above. The effects of family doctors, hospitals, and internet resources were higher for COVID-19 preventive behaviors of urban publics than for rural publics. Finally, the effect of internet resources on COVID-19 preventive behaviors of females was lower than males.

**Conclusions:**

Obtaining COVID-19-related information through internet resources had the most significant effect on COVID-19 preventive behaviors, but was not significant among publics with old age, low education, low income, and living in rural area.

## Introduction

COVID-19 is a new infectious disease with a strong transmission ability and has caused hundreds of millions of infections worldwide. The World Health Organization (WHO) has declared COVID-19 as a public health emergency of international concern, and WHO Information Network for Epidemics was launched to address the vast amounts of information being disseminated (1, 2). The COVID-19 pandemic requires large-scale behavior change to control virus spreading (3). The government issued COVID-19 preventive behavioral guidelines to the public, including wear masks, wash hands frequently, not agglomerate, and others (4). Despite the efforts of the government and related agencies, there are still some people who do not take protective behaviors against COVID-19.

Scientific information and knowledge are important to improve COVID-19 preventive behaviors for public. It is essential to help the public learn more about COVID-19 as soon as possible (5). During the COVID-19 pandemic, obtaining credible information from trusted information sources are helpful to reduce the public fear and stress when facing COVID-19 and stop the spread of rumors (6). Currently, there are numerous information for the public, and some of them are not scientific, misinformation about COVID-19 is a major threat to public health (7). For example, social media can be a vehicle to disseminate erroneous, alarmists, and exaggerated information (8). And the dissemination of these misinformation can affect public COVID-19 preventive behaviors, which can lead to an increased risk of infection (9).

Social media such as internet and WeChat are the main sources to obtain COVID-19-related information in China, whereas health professionals, academic institutions, and governments were trusted sources of information (10, 11). Several studies indicated that different information sources had different effects on public psychological health regarding COVID-19. People who obtained COVID-19-related information through the internet, traditional media, and friends presented a higher current worry (12), while receiving information from medical staff was positively related with psychological wellbeing (13). However, the existing studies mainly focused on the effect of information sources on public psychology and risk perception, while their effects on public COVID-19 prevention behaviors also deserves to be studied (14, 15). Meanwhile, different publics often have different primary information sources. For example, females and higher income groups are more likely to select doctors or healthcare providers as their first source of health information than males and lower-income groups (16). Younger people prefer to obtain information through the internet, while older people prefer to use traditional media (12). Therefore, there may be differences in the effects of different information sources on COVID-19 prevention behaviors of different publics.

We conducted a nationwide network survey among Chinese citizens to evaluate the influence of different information sources on COVID-19 preventive behaviors, and to identify differences between publics. We hypothesized that information sources would have the effects on COVID-19 preventive behaviors and there would be differences across different publics. The results can help governments and related agencies to provide more scientific and accurate COVID-19-related information for different publics, which will improve public COVID-19 preventive behaviors and reduce the risk of COVID-19 infection.

## Materials and methods

### Study design and participants

We conducted a nationwide network survey among Chinese citizens from January 30, 2020 to February 20, 2020. We recruited university students as investigators from around the country, ensuring 1–3 investigators for each province-level region. Students from Taiwan Province were not recruited. All investigators were trained uniformly through the internet. Owing to the impact of the closed-off management, the communities where the investigators live were used as the investigation sites. The investigators randomly selected families in the community and, with the people's informed consent, sent the electronic questionnaires to these families through an online survey platform (SurveyStar: Changsha Ranxing Science and Technology). Members of the selected family who were 16 years and above, without cognitive impairment, without serious mental illness, and voluntarily participated in the survey could answer the questionnaire. For people who do not use electronic questionnaire, they could complete questionnaire with the help of other family members, or have the investigator complete the survey on them over the telephone. In order to ensure the quality of the questionnaire, investigators would check questionnaires at the end of the day's survey, and would confirm and verify unclear or incomplete answers by contacting participants. A total of 11,190 participants from 33 province-level regions (except Taiwan Province) were involved in this study. Regarding the participants, the age ranged from 16 to 67 years. 6,697 were females and 4,493 were males, 7,294 lived in urban area and 3,896 lived in rural area.

Province-level regions were categorized into different risk levels based on the number of COVID-19 confirmed cases on February 12, 2020, obtained from the National Health Commission of the People's Republic of China (17). Hubei Province was assessed as high-risk area (level-1). In addition, the number of confirmed cases in other provincial-level regions were ranked from largest to smallest, and the data were divided into 3 levels according to the method of quartile. The results showed that level-1 risk area including Hubei Province, level-2 risk area including 6 province-level regions, level-3 risk area including 17 province-level regions, and level-4 risk area including 10 province-level regions.

### Measures

#### COVID-19 preventive behaviors

In this survey we included 10 COVID-19 preventive behaviors, including (1) Wearing protective masks; (2) Covering your mouth and nose with a tissue when coughing or sneezing; (3) Washing hands carefully; (4) Indoor ventilation; (5) A healthy diet; (6) Avoiding eat bushmeat; (7) Health surveillance; (8) Avoiding contact with people with symptoms of respiratory diseases; (9) Avoiding crowds; (10) Avoiding visit their relatives and friends. For each behavior, participants were asked “which stage is your behavior?”. There were five stages can be selected, including pre-intention (I do not plan to take this behavior), interruption (I carried out this behavior, but now I stop), intention (I realized the importance of this behavior), planning (I have the plan of taking this behavior), and action (I carry out this behavior). The five stages were recorded as 0, 1, 2, 3, and 4 points, respectively. COVID-19 preventive behaviors scores ranged from 0 to 40, with higher scores indicating better COVID-19 preventive behaviors. Cronbach's α for this section was 0.958.

#### Information sources

The question “During the COVID-19 pandemic, when you encounter health problems or need health information, how do you get help? (You should choose any that apply)” was used to collect information sources of participants. Answers including: (1) 12320 telephone (National Public Health Hotline); (2) Acquaintances consulting; (3) Family doctors; (4) Hospitals; (5) Community health centers; (6) Internet resources (including social media, internet diagnosis and treatment platforms).

#### Covariates

Several sociodemographic characteristics were collected, including sex (female, male), age (25 and below, 26–40, 41–50, 51–60, 60 above), residence (rural, urban), income (no income, 1,000 below, 1,000–2,999, 3,000–4,999, 5,000 and above), education (non-educated, primary education, secondary education, higher education, and graduate education), smoke (non-smoker, current and former smoker), and drink (non-drinker, current and former drinker).

### Statistical analysis

Continuous variables were described as means ± standard deviations. Categorical variables were summarized as the counts and percentages in each category. The *X*^2^-test was used for categorical variables. A mixed linear model was used to analyze risk factors of COVID-19 preventive behaviors. Provincial units were used as clustering units to account for a within-clustering correlation attributable to the complex sample. Candidate related factors included sex, age, residence, income, education, smoke, and drink. All analyses were weighted by the distribution of sex and age ranked on a national survey (The Sixth National Census). The effects of different information sources on COVID-19 preventive behaviors of different publics were analyzed. The mean difference along with the 95% confidence interval were reported. The significant level was set up *P* < 0.05. All analyses were conducted with SPSS 24.0.

## Results

### Adoption of COVID-19 preventive behaviors

For the 10 COVID-19 preventive behaviors evaluated, the top three of them with the higher action rates were avoiding eat bushmeat (76.1%), a healthy diet (74.8%), and avoiding contact with people with symptoms of respiratory diseases (73.0%) (Table 1). The publics who were female, aged 26–40, lived in urban area, earned 5,000 yuans and above, had graduate education, and were non-smokers and non-drinkers had higher action rates on COVID-19 preventive behaviors. The adoption of four COVID-19 preventive behaviors (health surveillance, avoiding contact with people with symptoms of respiratory diseases, avoiding crowds, and avoiding visit their relatives and friends) had significant differences in different COVID-19 risk areas (*P* < 0.05) (Appendix).

**Table 1:** COVID-19 preventive behaviors by stages.

**Behaviors**	**Behavior stages [*****N*** **(%)]**				
	**Pre-intention**	**Interruption**	**Intention**	**Planning**	**Action**
1. Wearing protective masks	165 (1.5%)	351 (3.1%)	1,501 (13.4%)	1,375 (12.3%)	7,798 (69.7%)
2. Covering your mouth and nose with a tissue when coughing or sneezing	126 (1.1%)	277 (2.5%)	1,625 (14.5%)	1,355 (12.1%)	7,807 (69.8%)
3. Washing hands carefully	112 (1.0%)	323 (2.9%)	1,459 (13.0%)	1,400 (12.5%)	7,896 (70.6%)
4. Indoor ventilation	107 (1.0%)	305 (2.7%)	1,468 (3.1%)	1,357 (12.1%)	7,953 (71.1%)
5. A healthy diet	94 (0.8%)	187 (1.7%)	1,348 (12.0%)	1,192 (10.7%)	8,369 (74.8%)
6. Avoiding eat bushmeat	143 (1.3%)	161 (1.4%)	1,276 (11.4%)	1,097 (9.8%)	8,513 (76.1%)
7. Health surveillance	97 (0.9%)	238 (2.1%)	1,456 (13.0%)	1,491 (13.3%)	7,908 (70.7%)
8. Avoiding contact with people with symptoms of respiratory diseases	84 (0.8%)	216 (1.9%)	1,356 (12.1%)	1,363 (12.2%)	8,171 (73.0%)
9. Avoiding crowds	90 (0.8%)	325 (2.9%)	1,340 (12.0%)	1,339 (12.0%)	8,096 (72.3%)
10. Avoiding visit their relatives and friends	96 (0.8%)	273 (2.4%)	1,361 (12.2%)	1,362 (12.2%)	8,098 (72.4%)

### Risk factors for COVID-19 preventive behaviors

**Figure 1:**
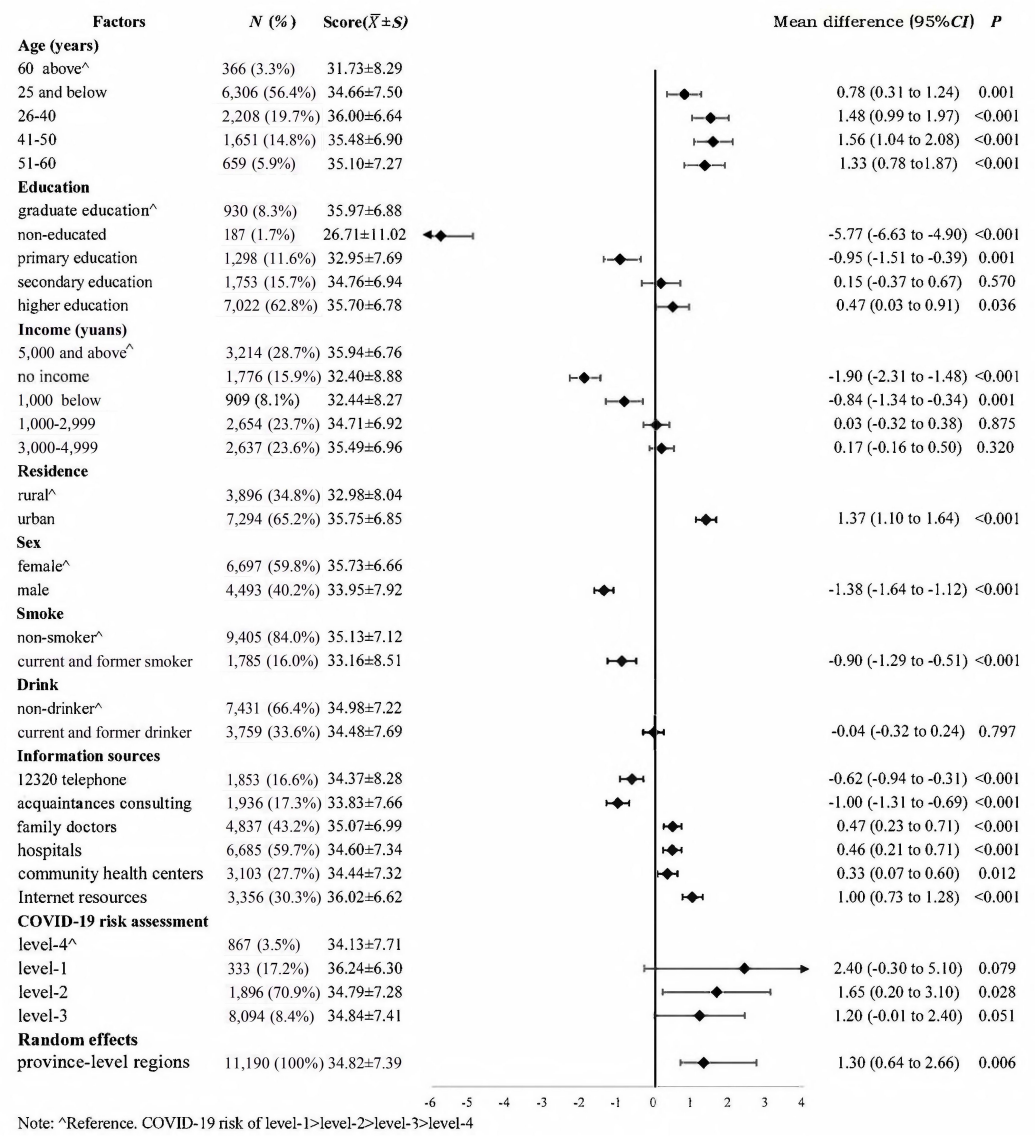
Risk factors for COVID-19 preventive behaviors.

Figure 1 presents the COVID-19 preventive behaviors scores of different publics and the effects of different factors for COVID-19 preventive behaviors. The COVID-19 risk in different province-level regions was the random effect that varied across provincial units. The Intra-Class Correction (ICC) of the model was 0.027. The results showed that older adults had the lowest scores. Compared to those with graduate education, those with non-educated (−5.77, 95%CI: −6.63 to −4.90) presented the lowest scores. Those who had no income (−1.90, 95% CI: −2.31 to −1.48) and earned below 1,000 yuans (−0.84, 95% CI: −1.34 to −0.34) showed lower scores than others. Urban publics (1.37, 95%CI: 1.10–1.64) had higher scores than rural one. Males (−1.38, 95% CI: −1.64 to −1.12) scored lower than females. The scores of current and former smokers (−0.90, 95%CI: −1.29 to −0.51) was lower than non-smokers. The public that lived in the area with higher COVID-19 risk presented higher scores (level-1, 36.24 > level-3, 34.84 > level-2, 34.79 > level-4, 34.13).

### The association between information sources and COVID-19 preventive behaviors

Among the six information sources, internet resources, family doctors, hospitals, and community health centers were positively associated with COVID-19 preventive behaviors (1.00 vs. 0.47 vs. 0.46 vs. 0.33, *P* < 0.05) (Figure 1). Table 2 presents the effects of them on COVID-19 preventive behaviors of different publics. For older adults, accessing to COVID-19-related information through family doctors (1.38, 95% CI: 0.63–2.12) and community health centers (1.22, 95% CI: 0.48–1.97) was positively associated with COVID-19 preventive behaviors. For the non-educated, family doctors (6.08, 95% CI: 4.71–7.45) and community health centers (3.69, 95% CI: 2.32–5.05) had positive effects on their COVID-19 preventive behaviors, except for internet resources and hospitals. Family doctors (0.72, 95% CI: 0.29–1.15) and internet resources (0.97, 95% CI: 0.51–1.42) were positively associated with COVID-19 preventive behaviors among those earning 5,000 yuans and above. The effects of family doctors (0.36, 95% CI: 0.07–0.65), hospitals (0.43, 95% CI: 0.13–0.73), and internet resources (0.92, 95% CI: 0.60–1.23) on COVID-19 preventive behaviors of urban publics were higher than rural publics. The effect of internet resources (0.43, 95% CI: 0.09–0.77) on female COVID-19 preventive behaviors was lower than on male. These four information sources had positive association with COVID-19 preventive behaviors in all areas, except for family doctors in level-4 risk area (−0.98, 95% CI: −1.81 to −0.14).

**Table 2:** The association between different information sources and COVID-19 preventive behaviors of different publics.

**Factors**	**Information sources [Mean difference (95%CI)]**			
	**Family doctors**	**Hospitals**	**Community health centers**	**Internet resources**
**Age (years)**				
25 and below	0.50 (0.15 to 0.85)^b^	0.69 (0.32 to 1.05)^b^	0.40 (0.02 to 0.78)^a^	0.82 (0.45 to 1.20)^b^
26–40	0.07 (−0.43 to 0.57)	−0.22 (−0.72 to 0.28)	−0.26 (−0.84 to 0.33)	0.96 (0.40 to 1.48)^b^
41–50	0.01 (−0.66 to 0.69)	−0.29 (−0.96 to 0.38)	−0.44 (−1.21 to 0.34)	1.34 (0.58 to 2.10)^b^
51–60	−1.16 (−2.2 to −0.71)^b^	−0.15 (−0.91 to 0.60)	0.30 (−0.52 to 1.11)	0.13 (−0.76 to 1.02)
60 above	1.38 (0.63 to 2.12)^b^	−0.50 (−1.30 to 0.30)	1.22 (0.48 to 1.97)^b^	−0.77 (−1.84 to 0.30)
**Education**				
Non-educated	6.08 (4.71 to 7.45)^b^	1.16 (−0.25 to 2.56)	3.69 (2.32 to 5.05)^b^	−1.90 (−4.30 to 0.50)
Primary education	0.39 (−0.25 to 1.03)	0.07 (−0.59 to 0.73)	0.73 (0.09 to 1.37)^a^	0.25 (−0.58 to 1.08)
Secondary education	−0.45 (−1.06 to 0.16)	0.08 (−0.54 to 0.70)	−0.08 (−0.73 to 0.56)	1.69 (0.98 to 2.41)^b^
Higher education	−0.06 (−0.37 to 0.24)	0.17 (−0.15 to 0.48)	0.80 (−0.26 to 0.42)	0.66 (0.34 to 0.97)^b^
Graduate education	1.32 (0.53 to 2.12)^b^	0.04 (−0.76 to 0.83)	−0.25 (−1.27 to 0.77)	1.03 (0.22 to 1.83)^b^
**Income (yuans)**				
No income	0.51 (−0.17 to 1.14)	1.03 (0.39 to 1.68)^b^	0.89 (0.23 to 1.56)^b^	0.74 (0.05 to 1.44)^a^
1,000 below	0.87 (0.04 to 1.70)^a^	0.02 (−0.84 to 0.87)	0.09 (−0.73 to 0.92)	0.18 (−0.81 to 1.16)
1,000–2,999	−0.57 (−1.06 to −0.09)^a^	−0.19 (−0.69 to 0.31)	−0.04 (−0.55 to 0.48)	0.69 (0.14 to 1.23)^a^
3,000–4,999	0.08 (−0.40 to 0.56)	0.41 (−0.08 to 0.90)	0.16 (−0.37 to 0.70)	0.75 (0.23 to 1.28)^b^
5,000 and above	0.72 (0.29 to 1.15)^b^	−0.14 (−0.57 to 0.29)	0.36 (−0.15 to 0.86)	0.97 (0.51 to 1.42)^b^
**Residence**				
Rural	0.02 (−0.38 to 0.42)	−0.37 (−0.79 to 0.04)	0.51 (0.10 to 0.92)^a^	0.45 (−0.02 to 0.91)
Urban	0.36 (0.07 to 0.65)^a^	0.43 (0.13 to 0.73)^b^	0.11 (−0.23 to 0.44)	0.92 (0.60 to 1.23)^b^
**Sex**				
Female	0.31 (0.00 to 0.63)	0.02 (−0.30 to 0.34)	0.08 (−0.27 to 0.43)	0.43 (0.09 to 0.77)^a^
Male	0.16 (−0.20 to 0.51)	0.34 (−0.03 to 0.70)	0.49 (0.11 to 0.88)^a^	1.25 (0.85 to 1.66)^b^
**COVID-19 risk assessment**				
Level-1	1.87 (0.81 to 2.93)^b^	2.54 (1.39 to 3.69)^b^	2.86 (1.63 to 4.08)^b^	3.18 (2.01 to 4.35)^b^
Level-2	0.99 (0.31 to 1.67)^b^	2.09 (1.34 to 2.83)^b^	1.67 (0.95 to 2.39)^b^	1.51 (0.79 to 2.23)^b^
Level-3	0.79 (0.22 to 1.37)^b^	2.37 (1.69 to 3.04)^b^	1.52 (0.93 to 2.09)^b^	1.92 (1.37 to 2.48)^b^
Level-4	−0.98 (−1.81 to −0.14)^a^	2.08 (1.24 to 2.92)^b^	1.24 (0.36 to 2.11)^b^	1.02 (0.11 to 1.93)^a^

Interaction terms and random coefficients. All other fixed effects remain similarly to Figure 1. The model controls for sex, age, residence, income, education, smoke, and drink.

a *P* < 0.05.

b *P* < 0.01.

## Discussion

This study found that the Chinese public generally had good COVID-19 preventive behaviors, mean COVID-19 preventive behaviors score was 34.82 (total score: 40). Action rates for all COVID-19 preventive behaviors were above or near 70%. The top three COVID-19 preventive behaviors with the higher action rate were avoiding eat bushmeat (76.1%), a healthy diet (74.8%), and avoiding contact with people with symptoms of respiratory diseases (73.0%). This indicated that the Chinese public had a comprehensive understanding of the infection sources, pathogenesis and virulence of the virus, transmissibility, and risk factors for infection and disease progression of COVID-19. In addition, we found that publics with old age, low education, low income, living in rural area, male, current and former smoker presented worse COVID-19 preventive behaviors. Therefore, more attention should be paid to the COVID-19 preventive behaviors of these publics to reduce their risk of COVID-19 infection. To information sources, internet resources had the most significant effect on COVID-19 preventive behaviors, while each information source presented different effect on COVID-19 preventive behaviors of different publics.

We analyzed the association between information sources and COVID-19 preventive behaviors. We found that obtaining COVID-19-related information through internet resources had the most significant effect on COVID-19 preventive behaviors. The internet has advantages such as timely information release, wide coverage, and fast dissemination (18, 19). In response to public health emergencies, like the outbreak of COVID-19, the government can release related information, collect public opinion and reaction, and deal with rumors in a timely manner through internet (20). At the same time, the public could be advocated and guided to take good protective measures by releasing information through internet, for example, a variety of COVID-19-related knowledge and guidelines for public were issued by the National Health Commission of the People's Republic of China and Chinese Center for Disease Control and Prevention through their official websites, microblogs, WeChat, and other social media. This provided the possibility to take preventive behaviors for public. Due to the large number of medical personnel from across the country traveling to help Hubei Province during the COVID-19 pandemic, some areas experienced weakened medical services and public basic medical needs could not be met in a timely manner (21). At the same time, the public was inconvenient to go to hospitals for medical services due to the closed-off management. Therefore, the public turned to internet diagnosis and treatment platforms to obtain health services, such as Ali Health, Ping An Good Doctor, and other internet hospitals. Besides providing basic medical services to the public, internet diagnosis and treatment platforms also have functions such as COVID-19-related information release, symptom diagnosis, psychology health assessment, dispelling rumors, and purchase of epidemic prevention supplies during the COVID-19 pandemic (22). These internet diagnosis and treatment platforms could provide professional information for the public.

However, we did not analyze the relationship between different types and contents of information from internet resources and COVID-19 preventive behaviors in this study. Therefore, we could not understand the impact of professional and unprofessional information on COVID-19 preventive behaviors. Some misinformation may exist on the internet and the public lack sufficient ability to discern the information accuracy, leading to inappropriate COVID-19 preventive behaviors, such as panic shopping, buying medical supplies or drugs, and taking drugs without a medical prescription (23). But it is undeniable that in the face of public health emergencies, governments, medical experts, and other authoritative institutions or individuals can release COVID-19-related information to the public, disclose policy measures, and carry out health promotion and education through the internet promptly. The public can also take the initiative to obtain the needed health knowledge through a series of internet platforms to raise preventive awareness and related preventive behaviors.

While accessing COVID-19-related information through internet resources is an important way to improve public COVID-19 preventive behaviors, we further found that it is not effective for all members of the public. Accessing COVID-19-related information through family doctors and community health centers can improve COVID-19 preventive behaviors of older adults, rather than internet resources. This can be related to the fact that older adults prefer to obtain information through traditional media (12). Wang et al. also found that older adults more like to obtain health information from radio instead of internet (24). Additionally, it has also been found that older adults were skeptical for information released through the internet (25), which also affected them access to COVID-19-related information through the internet resources. In this study, we found that the public with higher income and higher education had better COVID-19 preventive behaviors and obtaining COVID-19-related information through internet resources did not have positive effect on improving COVID-19 preventive behaviors in low income and low education publics. Incomes and education were associated with people's healthy behaviors (26, 27). The low income and low education public tend to have lower socioeconomic status, the low socioeconomic status group is more likely to ignore health promotion behavior (28). Guo et al. found that education and income were positively associated with seeking of web-based information on COVID-19 (29). The low income and low education public may lack the ability and effective way to access COVID-19-related information through internet, and have difficulty learning related knowledge and identifying misinformation on internet. Internet resources also did not improve COVID-19 preventive behaviors of rural public. There is a digital divide between urban and rural areas (30). Compared to the urban public, the rural public is less likely to access information through internet. The income and education levels of rural public are generally lower than urban public, and the network construction in rural area is relatively poor than urban area, especially in remote area. These factors are not conducive to the rural public to obtain COVID-19-related information through internet resources to improve their COVID-19 preventive behaviors. In addition, we found that females had better COVID-19 preventive behaviors than males. This may be related to the fact that females have better lifestyles and more health literacy than males (31). However, internet resources had a more significant effect on COVID-19 prevention behaviors in males. Compared with females, males could be paying more attention to health-related information during the COVID-19 pandemic than before, leading to a significant improvement in their healthy behaviors.

## Limitations

Our findings should be considered with several limitations. Firstly, we performed a cross-sectional survey, which makes it impossible to build causal relationships between variables. Secondly, owing to the impact of closed-off management, data were collected online. Although we helped people who do not use electronic questionnaires by performing a telephone survey and matched the age and the sex of our sample to the national population, the generalizability of our sample may still be a limitation. Finally, we mainly focused on whether participants obtained information through internet resources, while other variables related to internet resources, such as the type and content of the information obtained, were not be considered. The same situation existed for other information sources, so we cannot know the quality of the information provided by each information source. Future studies should incorporate more comprehensive variables to further analyze the association between information sources and COVID-19 preventive behaviors.

## Conclusion

Internet resources had an important and positive role in improving COVID-19 preventive behaviors, and governments and related agencies should timely provide COVID-19-related information on internet. However, it is important to focus on publics with old age, low education, low income, and living in rural area who have difficulty obtaining COVID-19-related information through internet resources to improve their behaviors. Therefore, in addition to internet resources, traditional offline health promotion and education should be conducted through hospitals, community health centers, and other professional institutions and personnel. This would ensure these publics can access scientifically and valid information, and improve their COVID-19 preventive behaviors and reduce the risk of COVID-19 infection.

## Data availability statement

The raw data supporting the conclusions of this article will be made available by the authors, without undue reservation.

## Ethics statement

Written informed consent was obtained from the individual(s) for the publication of any potentially identifiable images or data included in this article.

## Author contributions

YW and CZ conceived and designed the study. MY, MW, and YL provided valuable advice and guidance for this study. BX, XZ, YX, FD, and SX cleaned data. BX, XZ, and YX conducted a statistical analysis of the data. BX, YW, XZ, and YL drafted and edited the manuscript. All authors have read and agreed to the published version of the manuscript.

## Funding

This work was supported by the Directive Project of Medical Scientific Research Foundation in Guangdong (Grant Number: C2020062); Special Research Project of Prevention and Control during COVID-19 Epidemic in Universities of Guangdong (Grant Number: 2020KZDZX1046); Guangdong Basic and Applied Basic Research Foundation (Grant Number: 2022A1515011591); Discipline Construction Project of Guangdong Medical University (Grant Number: 4SG22265G); and Key Laboratory Development Project for Philosophy and Social Sciences in Guangdong (Grant Number: G620369695).

## Conflict of interest

The authors declare that the research was conducted in the absence of any commercial or financial relationships that could be construed as a potential conflict of interest.

## Publisher's note

All claims expressed in this article are solely those of the authors and do not necessarily represent those of their affiliated organizations, or those of the publisher, the editors and the reviewers. Any product that may be evaluated in this article, or claim that may be made by its manufacturer, is not guaranteed or endorsed by the publisher.
