# Supplementary material for: Effects of information sources on public preventive behaviors in health emergencies: Evidence from a digital epidemiologic study during the COVID-19 pandemic
Source: Front Public Health. 2022 Oct 14;10:981649. doi: 10.3389/fpubh.2022.981649 (PMC9615916; doi:10.3389/fpubh.2022.981649)
Supplement: Supplementary file 1 [file Data_Sheet_1.doc]

**APPENDIX. Adoption of COVID-19 preventive behaviors of different publics**

**TABLE A1. Wearing protective masks**

| **Factors** | **Others** | **Action** | **Action rate** | ***X²*** | ***P*** |
| --- | --- | --- | --- | --- | --- |
| **Sex** |  |  |  | 156.39 | <0.001 |
| Female | 1,732 (51.1%) | 4,965 (63.7%) | 74.1% |  |  |
| Male | 1,660 (48.9%) | 2,833(36.3%) | 63.1% |  |  |
| **Age (years)** |  |  |  | 214.78 | <0.001 |
| 25 and below | 2,057 (60.6%) | 4,249 (54.5%) | 67.4% |  |  |
| 26-40 | 489 (14.4%) | 1,719 (22.0%) | 77.9% |  |  |
| 41-50 | 442 (13.0%) | 1,209 (15.5%) | 73.2% |  |  |
| 51-60 | 197 (5.8%) | 462 (5.9%) | 70.1% |  |  |
| 60 above | 207 (6.2%) | 159 (2.1%) | 43.4% |  |  |
| **Residence** | |  |  | 360.91 | <0.001 |
| Rural | 1,621 (47.8%) | 2,275 (29.2%) | 58.4% |  |  |
| Urban | 1,771 (52.2%) | 5,523 (70.8%) | 75.7% |  |  |
| **Income (yuans)** |  |  |  | 272.34 | <0.001 |
| No income | 749 (22.1%) | 1,027 (13.2%) | 57.8% |  |  |
| 1,000 below | 385 (11.4%) | 524 (6.7%) | 57.6% |  |  |
| 1,000-2,999 | 799 (23.6%) | 1,855 (23.8%) | 69.9% |  |  |
| 3,000-4,999 | 717 (21.1%) | 1,920 (24.6%) | 72.8% |  |  |
| 5,000 and above | 742 (21.8%) | 2,472 (31.7%) | 76.9% |  |  |
| **Education** |  |  |  | 262.73 | <0.001 |
| Non-educated | 129 (3.8%) | 58 (0.7%) | 31.0% |  |  |
| Primary education | 541 (15.9%) | 757 (9.7%) | 58.3% |  |  |
| Secondary education | 561 (16.5%) | 1,192 (15.3%) | 68.0% |  |  |
| Higher education | 1,953 (57.6%) | 5,069 (65.0%) | 72.2% |  |  |
| Graduate education | 208 (6.2%) | 722 (9.3%) | 77.6% |  |  |
| **Smoke** |  |  |  | 72.82 | <0.001 |
| Non-smoker | 2,699(79.6%) | 6,706 (86.0%) | 71.3% |  |  |
| Current and former smoker | 693(20.4%) | 1,092 (14.0%) | 61.2% |  |  |
| **Drink** |  |  |  | 20.73 | <0.001 |
| Non-drinker | 2,148 (63.3%) | 5,283 (67.7%) | 71.1% |  |  |
| Current and former drinker | 1,244 (36.7%) | 2,515 (32.3%) | 66.9% |  |  |
| **COVID-19 risk assessment** | |  |  | 5.88 | 0.118 |
| Level-1 | 81 (2.4%) | 252 (3.2%) | 75.7% |  |  |
| Level-2 | 582 (17.2%) | 1,314 (16.9%) | 69.3% |  |  |
| Level-3 | 2,466 (72.7%) | 5,628 (72.2%) | 69.5% |  |  |
| Level-4 | 263 (7.7%) | 604 (7.7%) | 69.7% |  |  |

**TABLE A2. Covering your mouth and nose with a tissue when coughing or sneezing**

| **Factors** | **Others** | **Action** | **Action rate** | ***X²*** | ***P*** |
| --- | --- | --- | --- | --- | --- |
| **Sex** |  |  |  | 137.90 | <0.001 |
| Female | 1,745 (51.6%) | 4,952 (63.4%) | 73.9% |  |  |
| Male | 1,638 (48.4%) | 2,855 (36.6%) | 63.5% |  |  |
| **Age (years)** |  |  |  | 176.16 | <0.001 |
| 25 and below | 2,003 (59.2%) | 4,303 (55.1%) | 68.2% |  |  |
| 26-40 | 497 (14.7%) | 1,711 (21.9%) | 77.5% |  |  |
| 41-50 | 481 (14.2%) | 1,170 (15.0%) | 70.9% |  |  |
| 51-60 | 201 (5.9%) | 458 (5.9%) | 69.5% |  |  |
| 60 above | 201 (6.0%) | 165 (2.1%) | 45.1% |  |  |
| **Residence** | |  |  | 184.24 | <0.001 |
| Rural | 1,492 (44.1%) | 2,404 (30.8%) | 61.7% |  |  |
| Urban | 1,891 (55.9%) | 5,403 (69.2%) | 74.1% |  |  |
| **Income (yuans)** |  |  |  | 208.07 | <0.001 |
| No income | 750 (22.2%) | 1,026 (13.1%) | 57.8% |  |  |
| 1,000 below | 340 (10.1%) | 569 (7.3%) | 62.6% |  |  |
| 1,000-2,999 | 773 (22.8%) | 1,881 (24.1%) | 70.9% |  |  |
| 3,000-4,999 | 748 (22.1%) | 1,889 (24.2%) | 71.6% |  |  |
| 5,000 and above | 772 (22.8%) | 2,442 (31.3%) | 76.0% |  |  |
| **Education** |  |  |  | 221.90 | <0.001 |
| Non-educated | 117 (3.5%) | 70 (0.9%) | 37.4% |  |  |
| Primary education | 539 (15.9%) | 759 (9.7%) | 58.5% |  |  |
| Secondary education | 580 (17.1%) | 1,173 (15.0%) | 66.9% |  |  |
| Higher education | 1,924 (56.9%) | 5,098 (65.3%) | 72.6% |  |  |
| Graduate education | 223 (6.6%) | 707 (9.1%) | 76.0% |  |  |
| **Smoke** |  |  |  | 68.62 | <0.001 |
| Non-smoker | 2,696 (79.7%) | 6,709 (85.9%) | 71.3% |  |  |
| Current and former smoker | 687 (20.3%) | 1,098(14.1%) | 61.5% |  |  |
| **Drink** |  |  |  | 11.43 | 0.001 |
| Non-drinker | 2,169 (64.1%) | 5,262 (67.4%) | 70.8% |  |  |
| Current and former drinker | 1,214 (35.9%) | 2,545 (32.6%) | 67.7% |  |  |
| **COVID-19 risk assessment** | |  |  | 4.69 | 0.196 |
| Level-1 | 85 (2.5%) | 248 (3.2%) | 74.5% |  |  |
| Level-2 | 591 (17.5%) | 1,305 (16.7%) | 68.8% |  |  |
| Level-3 | 2,437 (72.0%) | 5,657 (72.5%) | 69.9% |  |  |
| Level-4 | 270 (8.0%) | 597 (7.6%) | 68.9% |  |  |

**TABLE A3. Washing hands carefully**

| **Factors** | **Others** | **Action** | **Action rate** | ***X²*** | ***P*** |
| --- | --- | --- | --- | --- | --- |
| **Sex** |  |  |  | 139.76 | <0.001 |
| Female | 1,692 (51.4%) | 5,005 (63.4%) | 74.7% |  |  |
| Male | 1,602 (48.6%) | 2,891 (36.6%) | 64.3% |  |  |
| **Age (years)** |  |  |  | 134.76 | <0.001 |
| 25 and below | 1,982 (60.2%) | 4,324 (54.8%) | 68.6% |  |  |
| 26-40 | 511 (15.5%) | 1,697 (21.5%) | 76.9% |  |  |
| 41-50 | 437 (13.3%) | 1,214 (15.4%) | 73.5% |  |  |
| 51-60 | 182 (5.5%) | 477 (6.0%) | 72.4% |  |  |
| 60 above | 182 (5.5%) | 184 (2.3%) | 50.3% |  |  |
| **Residence** | |  |  | 241.79 | <0.001 |
| Rural | 1,504 (45.7%) | 2,392 (30.3%) | 61.4% |  |  |
| Urban | 1,790 (54.3%) | 5,504 (69.7%) | 75.5% |  |  |
| **Income (yuans)** |  |  |  | 214.50 | <0.001 |
| No income | 721 (21.9%) | 1,055 (13.4%) | 59.4% |  |  |
| 1,000 below | 348 (10.6%) | 561 (7.1%) | 61.7% |  |  |
| 1,000-2,999 | 779 (23.6%) | 1,875 (23.7%) | 70.6% |  |  |
| 3,000-4,999 | 709 (21.5%) | 1,928 (24.4%) | 73.1% |  |  |
| 5,000 and above | 737 (22.4%) | 2,477 (31.4%) | 77.1% |  |  |
| **Education** |  |  |  | 226.42 | <0.001 |
| Non-educated | 119 (3.6%) | 68 (0.9%) | 36.4% |  |  |
| Primary education | 520 (15.8%) | 778 (9.9%) | 59.9% |  |  |
| Secondary education | 563 (17.1%) | 1,190 (15.1%) | 67.9% |  |  |
| Higher education | 1,882 (57.1%) | 5,410 (65.1%) | 73.2% |  |  |
| Graduate education | 210 (6.4%) | 720 (9.0%) | 77.4% |  |  |
| **Smoke** |  |  |  | 60.71 | <0.001 |
| Non-smoker | 2,631 (79.9%) | 6,774 (85.8%) | 72.0% |  |  |
| Current and former smoker | 663 (20.1%) | 1,122 (14.2%) | 62.9% |  |  |
| **Drink** |  |  |  | 18.32 | <0.001 |
| Non-drinker | 2,090 (63.4%) | 5,341 (67.6%) | 71.9% |  |  |
| Current and former drinker | 1,204 (36.6%) | 2,555 (32.4%) | 68.0% |  |  |
| **COVID-19 risk assessment** | |  |  | 3.41 | 0.333 |
| Level-1 | 86 (2.6%) | 247 (3.1%) | 74.2% |  |  |
| Level-2 | 552 (16.8%) | 1,344 (17.0%) | 70.9% |  |  |
| Level-3 | 2,386 (72.4%) | 5,708 (72.3%) | 70.5% |  |  |
| Level-4 | 270 (8.2%) | 597 (7.6%) | 68.9% |  |  |

**TABLE A4. Indoor ventilation**

| **Factors** | **Others** | **Action** | **Action rate** | ***X²*** | ***P*** |
| --- | --- | --- | --- | --- | --- |
| **Sex** |  |  |  | 87.77 | <0.001 |
| Female | 1,717 (53.0%) | 4,980 (62.6%) | 74.4% |  |  |
| Male | 1,502 (47.0%) | 2,973 (37.4%) | 66.2% |  |  |
| **Age (years)** |  |  |  | 114.63 | <0.001 |
| 25 and below | 2,027 (62.6%) | 4,279 (53.8%) | 67.9% |  |  |
| 26-40 | 510 (15.8%) | 1,698 (21.4%) | 76.9% |  |  |
| 41-50 | 390 (12.0%) | 1,261 (15.9%) | 76.4% |  |  |
| 51-60 | 166 (5.1%) | 493 (6.2%) | 74.8% |  |  |
| 60 above | 144 (4.5%) | 222 (2.7%) | 60.7% |  |  |
| **Residence** | |  |  | 194.88 | <0.001 |
| Rural | 1,446 (44.7%) | 2,405 (30.8%) | 62.9% |  |  |
| Urban | 1,791 (55.3%) | 5,503 (69.2%) | 75.4% |  |  |
| **Income (yuans)** |  |  |  | 219.52 | <0.001 |
| No income | 728 (22.5%) | 1,048 (13.2%) | 59.0% |  |  |
| 1,000 below | 327 (10.1%) | 582 (7.3%) | 64.0% |  |  |
| 1,000-2,999 | 767 (23.7%) | 1,887 (23.7%) | 71.1% |  |  |
| 3,000-4,999 | 686 (21.2%) | 1,951 (24.5%) | 74.0% |  |  |
| 5,000 and above | 729 (22.5%) | 2,485 (31.3%) | 77.3% |  |  |
| **Education** |  |  |  | 137.47 | <0.001 |
| Non-educated | 106 (3.3%) | 81(1.0%) | 43.3% |  |  |
| Primary education | 471 (14.6%) | 827 (10.4%) | 63.7% |  |  |
| Secondary education | 549 (17.0%) | 1,204 (15.1%) | 68.7% |  |  |
| Higher education | 1,897 (58.6%) | 5,125 (64.4%) | 73.0% |  |  |
| Graduate education | 214 (6.5%) | 716 (9.0%) | 77.0% |  |  |
| **Smoke** |  |  |  | 41.87 | <0.001 |
| Non-smoker | 2,607 (80.5%) | 6,798 (85.5%) | 72.3% |  |  |
| Current and former smoker | 630 (19.5%) | 1,155 (14.5%) | 64.7% |  |  |
| **Drink** |  |  |  | 12.35 | <0.001 |
| Non-drinker | 2,070 (63.9%) | 5,361 (67.4%) | 72.1% |  |  |
| Current and former drinker | 1,167 (36.1%) | 2,592 (32.6%) | 69.0% |  |  |
| **COVID-19 risk assessment** | |  |  | 3.79 | 0.285 |
| Level-1 | 82 (2.5%) | 251 (3.2%) | 75.4% |  |  |
| Level-2 | 544 (16.8%) | 1,352 (17.0%) | 71.3% |  |  |
| Level-3 | 2,349 (72.6%) | 5,745 (72.2%) | 71.0% |  |  |
| Level-4 | 262 (8.1%) | 605 (7.6%) | 69.8% |  |  |

**TABLE A5. A healthy diet**

| **Factors** | **Others** | **Action** | **Action rate** | ***X²*** | ***P*** |
| --- | --- | --- | --- | --- | --- |
| **Sex** |  |  |  | 126.57 | <0.001 |
| Female | 1,435 (50.9%) | 5,262 (62.9%) | 78.6% |  |  |
| Male | 1,386 (49.1%) | 3,107 (37.1%) | 69.2% |  |  |
| **Age (years)** |  |  |  | 87.85 | <0.001 |
| 25 and below | 1,722 (61.0%) | 4,584 (54.8%) | 72.7% |  |  |
| 26-40 | 437 (15.5%) | 1,771 (21.2%) | 80.2% |  |  |
| 41-50 | 367 (13.0%) | 1,284 (15.3%) | 77.8% |  |  |
| 51-60 | 157 (5.6%) | 502 (6.0%) | 76.2% |  |  |
| 60 above | 138 (4.9%) | 228 (2.7%) | 62.3% |  |  |
| **Residence** | |  |  | 154.31 | <0.001 |
| Rural | 1,254 (44.5%) | 2,642 (31.6%) | 67.8% |  |  |
| Urban | 1,567 (55.5%) | 5,727 (68.4%) | 78.5% |  |  |
| **Income (yuans)** |  |  |  | 212.17 | <0.001 |
| No income | 667 (23.6%) | 1,109 (13.3%) | 62.4% |  |  |
| 1,000 below | 272 (9.6%) | 637 (7.6%) | 70.1% |  |  |
| 1,000-2,999 | 628 (22.3%) | 2,026 (24.2%) | 76.3% |  |  |
| 3,000-4,999 | 618 (21.9%) | 2,019 (24.1%) | 76.6% |  |  |
| 5,000 and above | 636 (22.6%) | 2,578 (30.8%) | 80.2% |  |  |
| **Education** |  |  |  | 170.18 | <0.001 |
| Non-educated | 98 (3.5%) | 89 (1.1%) | 47.6% |  |  |
| Primary education | 441 (15.6%) | 857 (10.2%) | 66.0% |  |  |
| Secondary education | 489 (17.3%) | 1,264 (15.1%) | 72.1% |  |  |
| Higher education | 1,617 (57.3%) | 5,405 (64.6%) | 77.0% |  |  |
| Graduate education | 176 (6.3%) | 754 (9.0%) | 81.1% |  |  |
| **Smoke** |  |  |  | 61.60 | <0.001 |
| Non-smoker | 2,239 (79.4%) | 7,166 (85.6%) | 76.2% |  |  |
| Current and former smoker | 582 (20.6%) | 1,203 (14.4%) | 67.4% |  |  |
| **Drink** |  |  |  | 14.06 | <0.001 |
| Non-drinker | 1,792 (63.5%) | 5,639 (67.4%) | 75.9% |  |  |
| Current and former drinker | 1,029 (36.4%) | 2,730 (32.6%) | 72.6% |  |  |
| **COVID-19 risk assessment** | |  |  | 2.57 | 0.463 |
| Level-1 | 75 (2.7%) | 258 (3.1%) | 77.5% |  |  |
| Level-2 | 478 (16.9%) | 1,418 (16.9%) | 74.8% |  |  |
| Level-3 | 2,035 (72.1%) | 6,059 (72.4%) | 74.9% |  |  |
| Level-4 | 233 (8.3%) | 634 (7.6%) | 73.1% |  |  |

**TABLE A6. Avoiding eat bushmeat**

| **Factors** | **Others** | **Action** | **Action rate** | ***X²*** | ***P*** |
| --- | --- | --- | --- | --- | --- |
| **Sex** |  |  |  | 115.87 | <0.001 |
| Female | 1,364 (51.0%) | 5,333 (62.6%) | 79.6% |  |  |
| Male | 1,313 (49.0%) | 3,180 (37.4%) | 70.8% |  |  |
| **Age (years)** |  |  |  | 64.85 | <0.001 |
| 25 and below | 1,596 (59.6%) | 4,710 (55.3%) | 74.7% |  |  |
| 26-40 | 419 (15.7%) | 1,789 (21.0%) | 81.0% |  |  |
| 41-50 | 374 (14.0%) | 1,277 (15.0%) | 77.3% |  |  |
| 51-60 | 158 (5.9%) | 501 (5.9%) | 76.0% |  |  |
| 60 above | 130 (4.9%) | 236 (2.8%) | 64.5% |  |  |
| **Residence** | |  |  | 176.92 | <0.001 |
| Rural | 1,218 (45.5%) | 2,678 (31.5%) | 68.7% |  |  |
| Urban | 1,459 (54.5%) | 5,835 (68.5%) | 80.0% |  |  |
| **Income (yuans)** |  |  |  | 201.92 | <0.001 |
| No income | 638 (23.8%) | 1,138 (13.4%) | 64.1% |  |  |
| 1,000 below | 264 (9.9%) | 645 (7.6%) | 71.0% |  |  |
| 1,000-2,999 | 589 (22.0%) | 2,065 (24.3%) | 77.8% |  |  |
| 3,000-4,999 | 551 (20.6%) | 2,086 (24.5%) | 79.1% |  |  |
| 5,000 and above | 635 (23.7%) | 2,579 (30.2%) | 80.2% |  |  |
| **Education** |  |  |  | 187.21 | <0.001 |
| Non-educated | 87 (3.2%) | 100 (1.2%) | 53.5% |  |  |
| Primary education | 447 (16.7%) | 851 (10.0%) | 65.6% |  |  |
| Secondary education | 475 (17.7%) | 1,278 (15.0%) | 72.9% |  |  |
| Higher education | 1,508 (56.3%) | 5,514 (64.8%) | 78.5% |  |  |
| Graduate education | 160 (6.1%) | 770 (9.0%) | 82.8% |  |  |
| **Smoke** |  |  |  | 65.73 | <0.001 |
| Non-smoker | 2,116 (79.0%) | 7,289 (85.6%) | 77.5% |  |  |
| Current and former smoker | 561 (21.0%) | 1,224 (14.4%) | 68.6% |  |  |
| **Drink** |  |  |  | 13.64 | <0.001 |
| Non-drinker | 1,699 (63.5%) | 5,732 (67.3%) | 77.1% |  |  |
| Current and former drinker | 978 (36.5%) | 2,781 (32.7%) | 74.0% |  |  |
| **COVID-19 risk assessment** | |  |  | 1.28 | 0.734 |
| Level-1 | 72 (2.7%) | 261 (3.1%) | 78.4% |  |  |
| Level-2 | 463 (17.3%) | 1,433 (16.8%) | 75.6% |  |  |
| Level-3 | 1,932 (72.2%) | 6,162 (72.4%) | 76.1% |  |  |
| Level-4 | 210 (7.8%) | 657 (7.7%) | 75.8% |  |  |

**TABLE A7.** **Health surveillance**

| **Factors** | **Others** | **Action** | **Action rate** | ***X²*** | ***P*** |
| --- | --- | --- | --- | --- | --- |
| **Sex** |  |  |  | 91.00 | <0.001 |
| Female | 1,739 (53.0%) | 4,958 (62.7%) | 74.0% |  |  |
| Male | 1,543 (47.0%) | 2,950 (37.3%) | 65.7% |  |  |
| **Age (years)** |  |  |  | 142.65 | <0.001 |
| 25 and below | 1,978 (60.3%) | 4,328 (54.7%) | 68.6% |  |  |
| 26-40 | 502 (15.3%) | 1,706 (21.6%) | 77.3% |  |  |
| 41-50 | 432 (13.2%) | 1,219 (15.4%) | 73.8% |  |  |
| 51-60 | 187 (5.7%) | 472 (6.0%) | 71.6% |  |  |
| 60 above | 183 (5.5%) | 183 (2.3%) | 50.0% |  |  |
| **Residence** | |  |  | 197.36 | <0.001 |
| Rural | 1,465 (44.6%) | 2,431 (30.7%) | 62.4% |  |  |
| Urban | 1,817 (55.4%) | 5,477 (69.3%) | 75.1% |  |  |
| **Income (yuans)** |  |  |  | 226.60 | <0.001 |
| No income | 728 (22.2%) | 1,048 (13.3%) | 59.0% |  |  |
| 1,000 below | 333 (10.1%) | 576 (7.3%) | 63.4% |  |  |
| 1,000-2,999 | 804 (24.5%) | 1,850 (23.4%) | 69.7% |  |  |
| 3,000-4,999 | 698 (21.3%) | 1,939 (24.5%) | 73.5% |  |  |
| 5,000 and above | 719 (21.9%) | 2,495 (31.5%) | 77.6% |  |  |
| **Education** |  |  |  | 260.41 | <0.001 |
| Non-educated | 123 (3.7%) | 64 (0.8%) | 34.2% |  |  |
| Primary education | 526 (16.0%) | 772 (9.8%) | 59.5% |  |  |
| Secondary education | 573 (17.5%) | 1,180 (14.9%) | 67.3% |  |  |
| Higher education | 1,855 (56.5%) | 5,167 (65.3%) | 73.6% |  |  |
| Graduate education | 205 (6.3%) | 725 (9.2%) | 78.0% |  |  |
| **Smoke** |  |  |  | 47.44 | <0.001 |
| Non-smoker | 2,637 (80.3%) | 6,768 (85.6%) | 72.0% |  |  |
| Current and former smoker | 645 (19.7%) | 1,140 (14.4%) | 63.9% |  |  |
| **Drink** |  |  |  | 16.18 | <0.001 |
| Non-drinker | 2,088 (63.6%) | 5,343 (67.6%) | 71.9% |  |  |
| Current and former drinker | 1,194 (36.4%) | 2,565 (32.4%) | 68.2% |  |  |
| **COVID-19 risk assessment** | |  |  | 7.85 | 0.049 |
| Level-1 | 79 (2.4%) | 254 (3.2%) | 76.3% |  |  |
| Level-2 | 563 (17.2%) | 1,333 (16.9%) | 70.3% |  |  |
| Level-3 | 2,364 (72.0%) | 5,730 (72.5%) | 70.8% |  |  |
| Level-4 | 276 (8.4%) | 591 (7.4%) | 68.2% |  |  |

**TABLE A8.** **Avoiding contact with people with symptoms of respiratory diseases**

| **Factors** | **Others** | **Action** | **Action rate** | ***X²*** | ***P*** |
| --- | --- | --- | --- | --- | --- |
| **Sex** |  |  |  | 109.47 | <0.001 |
| Female | 1,566 (51.9%) | 5,131 (62.8%) | 76.6% |  |  |
| Male | 1,453 (48.1%) | 3,040 (37.2%) | 67.7% |  |  |
| **Age (years)** |  |  |  | 114.38 | <0.001 |
| 25 and below | 1,794 (59.4%) | 4,512 (55.2%) | 71.6% |  |  |
| 26-40 | 488 (16.2%) | 1,720 (21.1%) | 77.9% |  |  |
| 41-50 | 394 (13.1%) | 1,257 (15.4%) | 76.1% |  |  |
| 51-60 | 172 (5,7%) | 487 (6.0%) | 73.9% |  |  |
| 60 above | 171 (5.6%) | 195 (2.3%) | 53.3% |  |  |
| **Residence** | |  |  | 171.46 | <0.001 |
| Rural | 1,344 (44.5%) | 2,552 (31.2%) | 65.5% |  |  |
| Urban | 1,675 (55.5%) | 5,619 (68.8%) | 77.0% |  |  |
| **Income (yuans)** |  |  |  | 214.35 | <0.001 |
| No income | 693 (23.0%) | 1,083 (13.3%) | 61.0% |  |  |
| 1,000 below | 296 (9.8%) | 613 (7.5%) | 67.4% |  |  |
| 1,000-2,999 | 719 (23.8%) | 1,935 (23.7%) | 72.9% |  |  |
| 3,000-4,999 | 636 (21.1%) | 2,001 (24.5%) | 75.9% |  |  |
| 5,000 and above | 675 (22.3%) | 2,539 (31.0%) | 79.0% |  |  |
| **Education** |  |  |  | 213.71 | <0.001 |
| Non-educated | 108 (3.6%) | 79 (1.0%) | 42.2% |  |  |
| Primary education | 486 (16.1%) | 812 (9.9%) | 62.6% |  |  |
| Secondary education | 528 (17.5%) | 1,225 (15.0%) | 69.9% |  |  |
| Higher education | 1,699 (56.3%) | 5,323 (65.1%) | 75.8% |  |  |
| Graduate education | 198 (6.0%) | 732 (9.0%) | 78.7% |  |  |
| **Smoke** |  |  |  | 62.04 | <0.001 |
| Non-smoker | 2,402 (79.6%) | 7,003 (85.7%) | 74.5% |  |  |
| Current and former smoker | 617 (20.4%) | 1,168 (14.3%) | 65.4% |  |  |
| **Drink** |  |  |  | 12.64 | <0.001 |
| Non-drinker | 1,926 (63.8%) | 5,505 (67.4%) | 74.1% |  |  |
| Current and former drinker | 1,093 (36.2%) | 2,666 (32.6%) | 70.9% |  |  |
| **COVID-19 risk assessment** | |  |  | 9.14 | 0.028 |
| Level-1 | 72 (2.4%) | 261 (3.2%) | 78.4% |  |  |
| Level-2 | 501 (16.6%) | 1,395 (17.1%) | 73.6% |  |  |
| Level-3 | 2,186 (72.4%) | 5,908 (72.3%) | 73.0% |  |  |
| Level-4 | 260 (8.6%) | 607 (7.4%) | 70.7% |  |  |

**TABLE** **A9. Avoiding crowds**

| **Factors** | **Others** | **Action** | **Action rate** | ***X²*** | ***P*** |
| --- | --- | --- | --- | --- | --- |
| **Sex** |  |  |  | 115.91 | <0.001 |
| Female | 1,602 (51.8%) | 5,095 (62.9%) | 76.1% |  |  |
| Male | 1,492 (48.2%) | 3,001 (37.1%) | 66.8% |  |  |
| **Age (years)** |  |  |  | 117.69 | <0.001 |
| 25 and below | 1,847 (59.7%) | 4,459 (55.1%) | 70.7% |  |  |
| 26-40 | 504 (16.3%) | 1,704 (21.0%) | 77.2% |  |  |
| 41-50 | 399 (12.9%) | 1,252 (15.5%) | 75.8% |  |  |
| 51-60 | 170 (5.5%) | 489 (6.0%) | 74.2% |  |  |
| 60 above | 174 (5.6%) | 192 (2.4%) | 52.5% |  |  |
| **Residence** | |  |  | 139.03 | <0.001 |
| Rural | 1,343 (43.4%) | 2,553 (31.5%) | 65.5% |  |  |
| Urban | 1,751 (56.6%) | 5,543 (68.5%) | 76.0% |  |  |
| **Income (yuans)** |  |  |  | 208.40 | <0.001 |
| No income | 708 (22.9%) | 1,068 (13.2%) | 60.1% |  |  |
| 1,000 below | 307 (9.9%) | 602 (7.4%) | 66.2% |  |  |
| 1,000-2,999 | 718 (23.2%) | 1,936 (23.9%) | 72.9% |  |  |
| 3,000-4,999 | 638 (20.6%) | 1,999 (24.7%) | 75.8% |  |  |
| 5,000 and above | 723 (23.4%) | 2,491 (30.8%) | 77.5% |  |  |
| **Education** |  |  |  | 214.97 | 0.001 |
| Non-educated | 112 (3.6%) | 75 (0.9%) | 40.1% |  |  |
| Primary education | 496 (16.0%) | 802 (9.9%) | 61.8% |  |  |
| Secondary education | 525 (17.0%) | 1,228 (15.2%) | 70.1% |  |  |
| Higher education | 1,761 (56.9%) | 5,261 (65.0%) | 74.9% |  |  |
| Graduate education | 200 (6.5%) | 730 (9.0%) | 78.5% |  |  |
| **Smoke** |  |  |  | 69.53 | <0.001 |
| Non-smoker | 2,456 (79.4%) | 6,949 (85.8%) | 73.9% |  |  |
| Current and former smoker | 638 (20.6%) | 1,147 (14.2%) | 64.3% |  |  |
| **Drink** |  |  |  | 28.19 | <0.001 |
| Non-drinker | 1,936 (62.6%) | 5,495 (67.9%) | 73.9% |  |  |
| Current and former drinker | 1,158 (37.4%) | 2,601 (32.1%) | 69.2% |  |  |
| **COVID-19 risk assessment** | |  |  | 12.18 | 0.007 |
| Level-1 | 72 (2.3%) | 261 (3.2%) | 78.4% |  |  |
| Level-2 | 526 (17.0%) | 1,370 (16.9%) | 72.3% |  |  |
| Level-3 | 2,224 (71.9%) | 5,870 (72.5%) | 72.5% |  |  |
| Level-4 | 272 (8.8%) | 595 (7.4%) | 68.6% |  |  |

**TABLE A10.** **Avoiding visit their relatives and friends**

| **Factors** | **Others** | **Action** | **Action rate** | ***X²*** | ***P*** |
| --- | --- | --- | --- | --- | --- |
| **Sex** |  |  |  | 111.18 | <0.001 |
| Female | 1,606 (51.9%) | 5,091 (62.9%) | 76.0% |  |  |
| Male | 1,486 (48.1%) | 3,007 (37.1%) | 66.9% |  |  |
| **Age (years)** |  |  |  | 128.51 | <0.001 |
| 25 and below | 1,865 (60.3%) | 4,441 (54.8%) | 70.4% |  |  |
| 26-40 | 491 (15.9%) | 1,717 (21.2%) | 77.8% |  |  |
| 41-50 | 390 (12.6%) | 1,261 (15.6%) | 76.4% |  |  |
| 51-60 | 173 (5.6%) | 486 (6.0%) | 73.7% |  |  |
| 60 above | 173 (5.6%) | 193 (2.4%) | 52.7% |  |  |
| **Residence** | |  |  | 167.29 | <0.001 |
| Rural | 1,368 (44.2%) | 2,528 (31.2%) | 64.9% |  |  |
| Urban | 1,724 (55.8%) | 5,570 (68.8%) | 76.4% |  |  |
| **Income (yuans)** |  |  |  | 248.81 | <0.001 |
| No income | 731 (23.6%) | 1,045 (12.9%) | 58.8% |  |  |
| 1,000 below | 306 (9.9%) | 603 (7.4%） | 66.3% |  |  |
| 1,000-2,999 | 718 (23.2%) | 1,936 (23.9%) | 72.9% |  |  |
| 3,000-4,999 | 629 (20.3%) | 2,008 (24.8%) | 76.1% |  |  |
| 5,000 and above | 708 (23.0%) | 2,506 (31.0%) | 78.0% |  |  |
| **Education** |  |  |  | 228.70 | <0.001 |
| Non-educated | 113 (3.7%) | 74 (0.9%) | 39.6% |  |  |
| Primary education | 495 (16.0%) | 803 (9.9%) | 61.9% |  |  |
| Secondary education | 544 (17.6%) | 1,209 (14.9%) | 69.0% |  |  |
| Higher education | 1,741 (56.3%) | 5,281 (65.2%) | 75.2% |  |  |
| Graduate education | 199 (6.4%) | 731 (9.0%) | 78.6% |  |  |
| **Smoke** |  |  |  | 59.65 | <0.001 |
| Non-smoker | 2,465 (79.7%) | 6,940 (85.7%) | 73.8% |  |  |
| Current and former smoker | 627 (20.3%) | 1,158 (14.3%) | 64.9% |  |  |
| **Drink** |  |  |  | 20.16 | <0.001 |
| Non-drinker | 1,953 (63.2%) | 5,478 (67.6%) | 73.7% |  |  |
| Current and former drinker | 1,139 (36.8%) | 2,620 (32.4%) | 69.7% |  |  |
| **COVID-19 risk assessment** | |  |  | 10.86 | 0.013 |
| Level-1 | 69 (2.2%) | 264 (3.3%) | 79.3% |  |  |
| Level-2 | 533 (17.2%) | 1,363 (16.8%) | 71.9% |  |  |
| Level-3 | 2,229 (72.1%) | 5,865 (72.4%) | 72.5% |  |  |
| Level-4 | 261 (8.5%) | 606 (7.5%) | 69.9% |  |  |
